# Tuberculosis Outbreak in Marijuana Users, Seattle, Washington, 2004

**DOI:** 10.3201/eid1207.051436

**Published:** 2006-07

**Authors:** John E. Oeltmann, Eyal Oren, Maryam B. Haddad, Linda K. Lake, Theresa A. Harrington, Kashef Ijaz, Masahiro Narita

**Affiliations:** * Centers for Disease Control and Prevention, Atlanta, Georgia, USA;; † Public Health–Seattle and King County Tuberculosis Control Program, Seattle, Washington, USA;; ‡ University of Washington, Seattle, Washington, USA

**Keywords:** Tuberculosis, pulmonary, Outbreaks, Marijuana smoking, Dispatch

## Abstract

Matching *Mycobacterium tuberculosis* isolates were noted among 11 young tuberculosis patients socially linked through illicit drug–related activities. A large proportion of their friends, 14 (64%) of 22, had positive tuberculin skin-test results. The behavior of "hotboxing" (smoking marijuana inside a closed car with friends to repeatedly inhale exhaled smoke) fueled transmission.

Although overall US tuberculosis (TB) rates are declining, certain populations such as the foreign-born (*1**,**2*), homeless persons (*3**,**4*), and those who use illicit drugs (*5**,**6*) continue to challenge TB control efforts. A cluster of TB cases was recognized in Seattle from February to April 2004 among 4 young East-African immigrants with histories of incarceration and illicit drug use. Because patients resisted revealing names of contacts, traditional TB control efforts were hampered. We describe an outbreak fueled by illicit drug use and characterized by accelerated progression of disease.

## The Study

*Mycobacterium tuberculosis* isolates from all culture-positive TB patients in Seattle and King County, Washington, during 2003–2004 were genotyped by spacer oligonucleotide typing and mycobacterial interspersed repetitive unit methods. We included patients who had an isolate that matched the outbreak strain or who had a social link to an already included patient.

Patient medical records were reviewed, and infectious periods were calculated. For sputum smear–positive patients, the infectious period extended from 3 months before symptom onset or the first positive smear (whichever was earlier) until 2 weeks after the start of appropriate TB treatment or until the patient was placed into isolation or produced consecutively negative smears. For sputum smear–negative patients, the infectious period extended from 1 month before symptom onset, the start of appropriate TB treatment, or the date that the patient was isolated (whichever was earlier), until 2 weeks after the start of appropriate TB treatment or until patient isolation (*7*).

We interviewed patients to learn their contacts, activities, and locations frequented while they were contagious. Additional contacts were found by outreach workers and a disease intervention specialist from the East-African community who was hired to work in the neighborhoods frequented by the patients. While in these neighborhoods, outreach workers and the disease intervention specialist recruited persons seen with patients or their contacts to be evaluated for TB and latent TB infection. Contact activities, specifically those related to illicit drugs, were observed or self-reported.

We categorized contacts as friends or others. Friends were defined as contacts of patients who spent time within a close-knit network of young men who exhibited similar marijuana-using behavior. Other contacts were defined as the families and relatives of patients and those who were named but were not closely associated with this network. Contacts received a TB evaluation including a tuberculin skin test (TST) to detect infection. Infection rates for friends and others were compared to guide contact prioritization for screening.

Patient 1 was first evaluated in December 2003, when a chest radiograph suggested pulmonary TB (i.e., upper lobe cavitary infiltrate). However, only clarithromycin was prescribed, and the patient was lost to follow-up. He was again seen in an emergency room in April 2004 after the infection evolved into bilateral extensive pulmonary TB. His sputum tested smear-positive for acid-fast bacilli. He was reluctant to name contacts.

Ten additional patients were found from February to October 2004 (Table 1). Isolates from all patients had matching TB genotypes. In Washington State, this genotype has only been identified among the patients in this outbreak. Patients' median age was 22 years (range 18–41). Eight patients were born in East Africa; a median of 13 years (range 6–22) had passed since their arrival in the United States. All but 1 patient were of East-African origin. Patient 5 was a white woman who received illicit drugs from patient 1.

**Table 1:** Tuberculosis outbreak patient and disease characteristics, N = 11

Characteristic	n
Patient	
East African origin	10
Foreign birth	8
Male	9
Incarceration history	11
Recent victim of assault	7
Illicit drug use	11
Hotboxing	11
Unemployed	11
Disease	
Pulmonary disease	11
Cavitary	7
Culture-confirmed	11
Sputum smear-positive for acid-fast bacilli	8
Symptomatic at diagnosis	9
HIV infection*	1

*Unknown for 1 patient.

Patients were symptomatic and had findings indicating infectiousness: all had pulmonary TB, 7 had cavitary disease, and 8 had sputum that tested smear-positive for acid-fast bacilli. One patient was HIV infected. Consecutive chest radiographs indicated progression to cavitary disease in <75 days weeks in 3 patients and <121 days in another patient. Table 2 shows the dates of clear chest radiographs interpreted as normal and the first chest radiographs showing disease.

**Table 2:** Chest radiograph dates and results, N = 11

Patient	HIV infection	Date of normal chest radiograph before TB diagnosis	Date of first abnormal chest radiograph consistent with TB	No. days between normal and abnormal chest radiographs	Cavitary disease
1*	Declined	Undocumented	12/24/2003		Yes
2	Negative	Undocumented	2/22/2004		No
3	Negative	2/7/2004	4/19/2004	72	Yes
4	Negative	2/10/2004	4/25/2004	75	Yes
5	Positive	1/13/2004	5/13/2004	121	No
6	Negative	Undocumented	6/18/2004		Yes
7	Negative	5/15/2004	6/24/2004	40	Yes
8	Negative	Undocumented	7/9/2004		No
9	Negative	8/17/2003	7/23/2004	341	Yes
10	Negative	5/14/2003	8/30/2004	474	Yes
11	Negative	Undocumented	8/26/2004		No

*Source case.

While contagious, patients stayed in various locations, including cars, for most of the day. A single-bedroom apartment occupied by at least 1 patient while he was contagious was regularly visited by 2 other patients. Numerous members of the friend network slept there on any given night, and many others would regularly visit during a 10-week period beginning in April 2004 (Figure). The occupants nailed boards over the apartment windows to conceal activities, primarily marijuana use, from outsiders.

**Figure:**
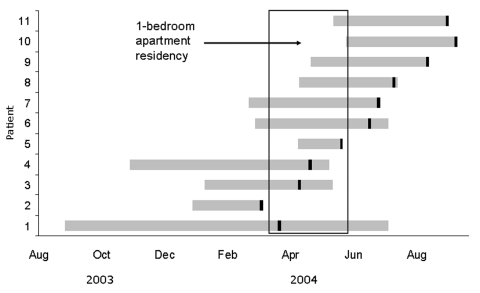
Infectious periods of tuberculosis patients. Vertical black bars indicate treatment start dates.

All patients were unemployed and had histories of incarceration and illicit drug use. No patients spent time together while incarcerated. All reported frequent "hotboxing," the practice of smoking marijuana with others in a vehicle with the windows closed so that exhaled smoke is repeatedly inhaled.

The Figure illustrates patients' infectious periods. Considerable overlap in infectious periods was noted, which highlights the potential for simultaneous contact with multiple contagious patients. We found 121 potentially exposed contacts. Fifty-four were friends, and the remaining were other contacts. At least 31 (57%) friend contacts spent time at the 1-bedroom apartment. After those with a past positive TST result were removed, 14 (64%) of 22 screened friends and 6 (23%) of 26 other contacts had a positive TST result. The risk for a positive TST result was 2.8× greater among friends than among other contacts (95% confidence interval = 1.3–6.0). Twenty-nine (54%) friend contacts self-reported or were observed hotboxing. Among the friends who reported or were observed hotboxing, 11 (79%) of 14 who received a TST had a positive result. Twelve friend contacts began treatment for latent TB infection, and 8 completed treatment.

## Conclusions

Risk factors for TB include birth in a country with high TB prevalence (*2*) and incarceration (*8*). Although most patients in this outbreak were foreign-born and had histories of incarceration, genotyping results and epidemiologic findings suggest that TB was transmitted recently in the community rather than before immigration or during incarceration.

Frequent marijuana use has been reported among TB outbreak patients (*9*) and was the behavior linking these patients together. Creative sharing of marijuana has been described recently as a factor for *M. tuberculosis* transmission. In Australia, sharing a water pipe (i.e., "bong") was linked to transmission (*10*). "Shotgunning" refers to inhaling smoke from illicit drugs then exhaling it directly into another's mouth (*11*) and was associated with *M. tuberculosis* transmission among a group of exotic dancers and their contacts (*12*).

This investigation noted that a similar activity, hotboxing, might have contributed to transmission. As with shotgunning, hotboxing promotes the sharing of exhaled smoke and air. One patient with smear-positive cavitary disease reported daily hotboxing with friends, often for most of the day. In addition, marijuana smoking might induce cough, creating an ideal environment for transmission. Many friends stayed and used marijuana at the single-bedroom apartment during the height of the outbreak. Furthermore, by nailing boards over the windows, ventilation was limited, creating an environment similar to that of hotboxing.

Disease rapidly progressed in HIV-negative patients in this outbreak. Seven patients had cavitary pulmonary TB. Three had chest radiographs interpreted as normal <75 days before TB diagnosis. Although progressive primary TB by nature is thought to be due to recent transmission, progressive primary TB with cavitation is uncommon (*13*). The pathogenesis of progressive primary TB with cavititation is not clear. However, frequent marijuana use and the setting of intense exposure may have played a role. In addition, poor nutrition and unhealthy lifestyles might have predisposed these young men to more rapid progression of disease. While no laboratory investigation to assess genetic susceptibility or strain virulence was conducted, these factors might have also contributed to the development of cases.

This outbreak resembles an outbreak reported among regular patrons of a neighborhood bar (*14*). Both were fueled by a highly infectious source patient who spent extended amounts of time indoors with 1 group of persons who regularly used substances (i.e., alcohol or marijuana). The result in both situations was a higher than expected incidence of TB disease and latent TB infection. In the outbreak reported in this article, however, the substance of choice was illicit and further complicated the control of this outbreak.

Patients' illicit drug activities promoted a reluctance to name contacts at risk and locations frequented. Traditional name- or location-based contact investigations did not work. Efforts had to revolve around meeting these young patients at times and locations convenient to the group. Then after gaining the groups' trust, outreach workers successfully found and screened contacts. Many successful screenings took place on street corners and in parking spaces throughout the community. Often outreach workers were successful only after spending hours driving throughout the community searching for patients and contacts. Four patients were originally screened as unnamed contacts located in the field. Alternative strategies to name-based contact investigations may become increasingly critical to TB control as TB recedes further from the general population, yet persists within smaller guarded groups (*15*).

## References

[1] Frieden TR, Fujiwara PI, Washko RM, Hamburg MA. Tuberculosis in New York City—turning the tide. N Engl J Med. 1995;333:229–33. 10.1056/NEJM199507273330406 7791840

[2] Talbot EA, Moore M, McCray E, Binkin NJ. Tuberculosis among foreign-born persons in the United States, 1993–1998. JAMA. 2000;284:2894–900. 10.1001/jama.284.22.2894 11147986

[3] Centers for Disease Control and Prevention. Tuberculosis transmission in a homeless shelter population—New York, 2002–2003. MMWR Morb Mortal Wkly Rep. 2005;54:149–51. 15716807

[4] Centers for Disease Control and Prevention. Tuberculosis outbreak among homeless persons—King County, Washington, 2002–2003. MMWR Morb Mortal Wkly Rep. 2003;52:1209–10. 14668713

[5] Leonhardt KK, Gentile F, Gilbert BP, Aiken M. A cluster of tuberculosis among crack house contacts in San Mateo County, California. Am J Public Health. 1994;84:1834–6. 10.2105/AJPH.84.11.1834 7977929 PMC1615218

[6] Centers for Disease Control and Prevention. Crack cocaine use among persons with tuberculosis—Contra Costa County, California, 1987–1990. MMWR Morb Mortal Wkly Rep. 1991;40:485–9. 2072883

[7] National Tuberculosis Controllers Association and CDC Advisory Group on Tuberculosis Genotyping. Guide to the application of genotyping to tuberculosis prevention and control. Atlanta: US Department of Health and Human Services; 2004.

[8] Bellin EY, Fletcher DD, Safyer SM. Association of tuberculosis infection with increased time in or admission to the New York City jail system. JAMA. 1993;269:2228–31. 10.1001/jama.1993.03500170058034 8474202

[9] Sterling TR, Thompson D, Stanley RL, McElroy PD, Madison A, Moore K, A multi-state outbreak of tuberculosis among members of a highly mobile social network: implications for tuberculosis elimination. Int J Tuberc Lung Dis. 2000;4:1066–73. 11092720

[10] Munckhof WJ, Konstantinos A, Wamsley M, Mortlock M, Gilpin C. A cluster of tuberculosis associated with use of a marijuana water pipe. Int J Tuberc Lung Dis. 2003;7:860–5. 12971670

[11] Perlman DC, Perkins MP, Paone D, Kochems L, Salomon N, Friedmann P, "Shotgunning" as an illicit drug smoking practice. J Subst Abuse Treat. 1997;14:3–9. 10.1016/S0740-5472(96)00182-1 9218230

[12] McElroy PD, Rothenberg RB, Varghese R, Woodruff R, Minns GO, Muth SQ, A network-informed approach to investigating a tuberculosis outbreak: implications for enhancing contact investigations. Int J Tuberc Lung Dis. 2003;7:S486–93. 14677842

[13] Barnes PF, Modlin RL, Ellner JJ. T-cell responses and cytokines. In: Bloom, BR, editor. Tuberculosis pathogenesis, protection, and control. Washington: American Society for Microbiology; 1994. p. 428.

[14] Kline SE, Hedemark LL, Davies SF. Outbreak of tuberculosis among regular patrons of a neighborhood bar. N Engl J Med. 1995;333:222–7. 10.1056/NEJM199507273330404 7791838

[15] Goldberg SV, Wallace J, Jackson CJ, Chaulk CP, Nolan CM. Cultural case management for latent tuberculosis infection. Int J Tuberc Lung Dis. 2004;8:76–82. 14974749

